# FED-UNet++: An Improved Nested UNet for Hippocampus Segmentation in Alzheimer’s Disease Diagnosis

**DOI:** 10.3390/s25165155

**Published:** 2025-08-19

**Authors:** Liping Yang, Wei Zhang, Shengyu Wang, Xiaoru Yu, Bin Jing, Nairui Sun, Tengchao Sun, Wei Wang

**Affiliations:** College of Computer Science and Technology, Changchun University, No. 6543, Satellite Road, Changchun 130022, China; 231502540@mails.ccu.edu.cn (W.Z.); 231502533@mails.ccu.edu.cn (S.W.); 231501508@mails.ccu.edu.cn (X.Y.); 231502527@mails.ccu.edu.cn (B.J.); 241501517@mails.ccu.edu.cn (N.S.); 241503550@mails.ccu.edu.cn (T.S.); wangwei@ccu.edu.cn (W.W.)

**Keywords:** Alzheimer’s disease, hippocampus segmentation, FED-UNet++, Residual Feature Reconstruction Block, Efficient Attention Pyramid, Dynamic Frequency Context Network

## Abstract

The hippocampus is a key structure involved in the early pathological progression of Alzheimer’s disease. Accurate segmentation of this region is vital for the quantitative assessment of brain atrophy and the support of diagnostic decision-making. To address limitations in current MRI-based hippocampus segmentation methods—such as indistinct boundaries, small target size, and limited feature representation—this study proposes an enhanced segmentation framework called FED-UNet++. The residual feature reconstruction block (FRBlock) is introduced to strengthen the network’s ability to capture boundary cues and fine-grained structural details in shallow layers. The efficient attention pyramid (EAP) module enhances the integration of multi-scale features and spatial contextual information. The dynamic frequency context network (DFCN) mitigates the decoder’s limitations in capturing long-range dependencies and global semantic structures. Experimental results on the benchmark dataset demonstrate that FED-UNet++ achieves superior performance across multiple evaluation metrics, with an IoU of 74.95% and a Dice coefficient of 84.43% ± 0.21%, outperforming the baseline model in both accuracy and robustness. These findings confirm that FED-UNet++ is highly effective in segmenting small and intricate brain structures like the hippocampus, providing a robust and practical tool for MRI-based analysis of neurodegenerative diseases.

## 1. Introduction

In the context of health-oriented human activity recognition (HAR) research, structural brain magnetic resonance imaging (MRI) can be regarded as a high-resolution sensing modality for acquiring precise anatomical data of the brain to monitor neurological health status. Prior studies have emphasized that high-quality MRI data, when combined with automated deep learning-based analytical pipelines, play a critical role in the early detection and continuous monitoring of neurodegenerative diseases. This perspective has been consistently underscored in numerous sensor-driven health monitoring and HAR studies [1]. In sensor-based biomedical analysis, the raw signals—represented in neuroimaging as high-resolution MRI scans—typically require feature extraction prior to computational modeling.

Traditional methods typically rely on handcrafted features derived from statistical, spatial, or frequency domains. In contrast, deep learning enables the automatic extraction of hierarchical deep neural representations directly from raw data, thereby laying the foundation for building more complex and expressive models. In recent years, the HAR field has not only emphasized performance within training distributions but has also placed increasing importance on domain generalization under varying scenarios and data distributions [2]. Prior studies have indicated that although deep models often exhibit strong performance within the training domain, handcrafted features may demonstrate greater robustness in cross-domain tasks. This phenomenon highlights the necessity of balancing representational capacity and generalization ability in model design. In light of these challenges, this study focuses on the accurate segmentation of the hippocampus—a critical structure for Alzheimer’s disease (AD) diagnosis.

Alzheimer’s disease (AD) is a common neurodegenerative disorder marked by progressive deterioration in memory, cognition, and behavior, substantially impairing patients’ quality of life [3,4]. Clinical studies have shown that the hippocampus—a brain region critical for learning and memory—is among the earliest and most severely affected structures in AD. Notably, hippocampal atrophy is closely associated with the early stages of AD and is widely recognized as a key imaging biomarker for diagnosis [4,5]. Therefore, accurate segmentation of the hippocampus on magnetic resonance imaging (MRI) is essential for quantifying brain atrophy and serves as a crucial step in the early auxiliary diagnosis of AD [6].

Recently, image segmentation techniques grounded in deep learning—especially those leveraging convolutional neural networks (CNNs)—have seen rapid development and achieved remarkable progress in the field of medical image analysis [7]. The classic U-Net architecture, with its symmetric encoder–decoder structure and skip connections, has demonstrated outstanding performance in various medical image segmentation tasks [8]. The improved variant U-Net++ introduces densely nested skip connections and multi-level feature fusion strategies, further enhancing segmentation accuracy in cases involving blurred boundaries and small organ structures such as the liver, heart, and brain regions [9]. U-Net++ offers advantages in feature fusion, making it particularly suitable for addressing the challenge of insufficient multi-scale semantic information, and is therefore widely applied to the segmentation of small, ill-defined structures such as the hippocampus.

To further enhance model performance, various improvement strategies have been integrated into the U-Net framework. For example, residual connections (residual blocks) have been introduced to improve feature propagation and alleviate gradient vanishing problems [10]; attention mechanisms such as SE, CBAM, and squeeze attention are used to enhance the model’s ability to focus on salient regions [11,12]; and the atrous spatial pyramid pooling (ASPP) module is adopted to extract contextual features at multiple spatial scales [13]. Moreover, recent studies have explored the incorporation of transformer architectures into medical image segmentation to improve global context modeling capability [14].

While the aforementioned methods partially address the local modeling limitations of U-Net++, several challenges persist. First, conventional convolutional modules in shallow layers lack sufficient nonlinear representational capacity, making it difficult to effectively capture edge structures and texture details. Second, many attention mechanisms focus solely on spatial or channel dimensions, without achieving efficient multi-scale contextual integration. Third, most existing frequency-domain modeling approaches are mostly limited to image enhancement or post-processing using Fourier transforms and have yet to be incorporated as learnable, frequency-aware mechanisms within end-to-end training frameworks. In particular, the absence of dynamic frequency modulation and multi-scale fusion in backbone modules limits the model’s ability to capture periodic patterns and long-range structural dependencies. To address these limitations, this study proposes an enhanced U-Net++-based framework for hippocampus segmentation, featuring three major innovations:

(1) A residual feature reconstruction block (FRBlock) is introduced to replace standard convolutional units. This module combines residual connections [15] with deep feature enhancement strategies to reinforce the network’s capacity to preserve boundary cues and structural details in shallow layers, thereby improving the semantic representation of low-level features and facilitating the accurate delineation of ambiguous hippocampal boundaries.

(2) An efficient attention pyramid (EAP) module is designed to enhance multi-scale structural modeling within the encoder. Considering the small size, complex anatomical structure, and contextual dependence of the hippocampus in MRI images, this module integrates the classical atrous spatial pyramid pooling (ASPP) module [13] with an efficient multi-scale attention (EMA) mechanism [16]. While ASPP provides multi-scale receptive fields, EMA guides the network’s attention toward semantically important regions. The EAP module effectively improves both structural feature integration and spatial contextual modeling, thereby enhancing the segmentation performance for fine-grained, multi-scale anatomical regions.

(3) A dynamic frequency context network (DFCN) is proposed and integrated into the deeper stages of the decoder for high-level semantic fusion. Inspired by the EDFFN module [17], DFCN aims to compensate for the decoder’s limited capacity to capture long-range dependencies and global contextual representations. It incorporates frequency-domain modeling and multi-scale fusion strategies to enhance the recognition of ambiguous boundaries and fine-grained structures such as the hippocampal tail. The introduction of DFCN enables the model to more effectively capture global semantics and periodic patterns, thereby significantly improving segmentation accuracy on high-resolution brain MRI scans.

To assess the performance of the proposed approach, comprehensive experiments were conducted on two widely recognized hippocampal MRI datasets: the publicly available Alzheimer’s hippocampus dataset from Kaggle [18] and the Task004_Hippocampus dataset provided by the Medical Segmentation Decathlon [19]. Experimental results demonstrate that the proposed method outperforms several state-of-the-art medical image segmentation models across multiple evaluation metrics, including IoU, Dice, recall, and precision, highlighting its robustness and generalizability.

It is worth noting that structural brain MRI not only provides rich spatial anatomical information but also serves as a valuable perceptual source for understanding neural function and health status. As a high-resolution imaging modality, MRI captures fine-grained data that can be used to monitor structural changes in the nervous system. The hippocampus, being one of the earliest affected regions in Alzheimer’s disease, exhibits morphological changes in MRI that can be effectively captured by perceptual models. Therefore, applying deep neural networks to brain image segmentation not only promotes automation in structural analysis but also supports the development of perceptual imaging-based monitoring systems for neurodegenerative diseases [20].

The structure of this paper is as follows: Section 2 reviews related work on medical image segmentation based on deep learning, with a particular emphasis on CNN-based approaches for hippocampus segmentation. Section 3 describes the architecture of the proposed FED-UNet++ model and its core components. Section 4 introduces the datasets, experimental settings, and evaluation metrics, followed by comprehensive result analysis and visualization. Finally, Section 5 concludes the paper and outlines potential directions for future research.

## 2. Related Works

Alzheimer’s disease (AD) is a prevalent neurodegenerative disorder, and early diagnosis is critical for slowing its progression. In recent years, deep learning-based medical image segmentation techniques have emerged as powerful tools for assisting in the diagnosis of AD. Among various brain structures, the hippocampus—essential for memory and learning—has been widely recognized as an early imaging biomarker owing to its progressive atrophy during the initial stages of the disease. Consequently, accurate and automated hippocampus segmentation has become a major focus of recent research [21].

### 2.1. Evolution of Deep Segmentation Networks

With the continuous improvement in the resolution and availability of medical imaging devices such as magnetic resonance imaging (MRI)—which can also be regarded as biosensors—deep learning has become the mainstream approach for medical image segmentation. In 2015, Long et al. first proposed the fully convolutional network (FCN) [22], which replaces traditional fully connected layers with convolutional layers, enabling the network to accept input images of arbitrary size and directly output pixel-level prediction maps that match the input dimensions. FCN pioneered the application of deep learning in the field of image segmentation. In 2015, Ronneberger et al. proposed the U-Net architecture [8], which was specifically designed for biomedical image segmentation.

This architecture adopts a symmetric encoder–decoder structure and introduces a skip connection mechanism that effectively preserves spatial information, significantly improving the segmentation accuracy of small organs and boundary structures in medical images. U-Net remains one of the most widely used networks in medical image segmentation tasks today. In 2017, Zhao et al. proposed the pyramid scene parsing network (PSPNet) [23], which introduced a multi-scale pyramid pooling module. This network integrates contextual information at different scales, effectively enhancing the model’s ability to understand various regions within the image. Chen et al. subsequently proposed the DeepLab series of models, among which DeepLabv3 [13] and DeepLabv3+ [24] adopted atrous convolution and the atrous spatial pyramid pooling (ASPP) module. These models also integrated a decoder structure to further enhance boundary recovery capabilities, demonstrating strong performance in extracting structures under complex backgrounds in medical images. Building upon the classic U-Net, Zhou et al. proposed UNet++ in 2018 [9], which incorporates dense skip connections and deep supervision mechanisms. This design enhances feature reuse and multi-scale fusion capabilities, thereby improving the network’s representation power and convergence speed. In recent years, the transformer architecture has been introduced into medical image segmentation. Chen et al. proposed TransUNet [14], which combines local features extracted by CNNs with global dependencies modeled by transformers. This approach significantly enhances the model’s ability to perceive structural information. Subsequently, Hatamizadeh et al. proposed UNETR (UNet with transformer encoder) [25], which utilizes a pure transformer encoder to extract semantic features from medical images. Swin-UNet [26] further introduced a hierarchical window-based attention mechanism, enhancing the model’s computational efficiency and multi-scale modeling capability.

### 2.2. The Application of Deep Learning Methods in Medical Image Segmentation

In recent years, with the development of deep learning, an increasing number of studies have focused on improving the accuracy and efficiency of medical image segmentation by introducing strategies such as multi-scale receptive fields and attention mechanisms. Wang et al. [27] proposed the MSTP-Net (multi-scale three-path network) model for retinal vessel segmentation. This network constructs three feature extraction paths with different scales, enabling effective fusion of spatial information across scales. As a result, the model enhances its ability to perceive fine-grained structures, highlighting the importance of multi-scale fusion in tasks involving precise structural segmentation. Zhao et al. [28] conducted a comprehensive review of deep learning-based cancer data fusion methods, covering strategies such as image-to-image, image-to-text, and image-to-genomics fusion. They emphasized the advantages of fusion mechanisms in improving diagnostic and segmentation performance. Huang et al. [29] proposed an improved U-Net for retinal vessel image segmentation, which enhances feature extraction capabilities through the introduction of residual modules. The model also incorporates cross-scale skip connections to improve multi-level information integration. This method outperformed the traditional U-Net and several of its variants across multiple datasets, validating the effectiveness of residual structures in modeling fine-grained targets such as microvessels. Xing et al. [30] proposed the FFTNet model, which incorporates the fast Fourier transform (FFT) into the medical image segmentation framework. By modeling structural features in the frequency domain and fusing them with spatial domain information, the model significantly improves robustness and segmentation accuracy in scenarios involving low-frequency deformations and structural degradation.

Meanwhile, to enhance the efficiency of model deployment in clinical practice and improve feature modeling capabilities, researchers have explored various lightweight design strategies. Hayat et al. [31] proposed the Attention GhostUNet++ network, which introduces channel, spatial, and depth attention mechanisms based on the GhostUNet++ backbone. This design enables multi-dimensional feature modeling and refinement, effectively improving segmentation accuracy while significantly reducing computational cost. The results validate the practicality and generalizability of lightweight attention mechanisms in complex structural segmentation tasks. Moreover, with the growing demand for modeling fine structures in medical images—such as vascular edges and tissue textures—frequency-domain modeling and multi-scale attention mechanisms have attracted increasing attention. In a review on endoscopic image super-resolution, Hayat et al. [32] systematically summarized research progress on traditional methods and deep learning techniques aimed at improving endoscopic image quality. The study focused on the role of super-resolution technologies in enhancing edge details and restoring textures, highlighting their importance for structural visualization and diagnostic accuracy in minimally invasive surgery. The review pointed out that introducing frequency modeling and structure-aware mechanisms holds promise for enhancing downstream tasks such as segmentation while improving image quality.

Therefore, segmentation strategies that integrate lightweight attention mechanisms, multi-scale modeling, and frequency-domain information fusion have become a key pathway for fine-grained structural modeling. These strategies also provide the theoretical foundation and practical support for the design of the proposed FED-UNet++ architecture in this study.

### 2.3. CNN-Based Methods for Hippocampus Segmentation

Recent advances in deep learning have substantially improved the performance of hippocampal segmentation methods. Liang et al. [33] introduced an integrated framework that combines vision transformer (ViT) with the U-Net++ model, leveraging the global modeling capacity of ViT and the multi-scale feature fusion capability of U-Net++ to better capture complex boundary structures. Helaly et al. [34] developed two enhanced U-Net variants for early detection of Alzheimer’s disease: SHPT-Net improved model practicality by reducing hyperparameter tuning complexity, while RESU-Net enhanced feature extraction through the integration of ResNet modules. Sanjay et al. [35] further integrated brain atrophy analysis with hippocampus segmentation and proposed KLW-RU-Net, which incorporates a Kullback–Leibler divergence-based regularization term into the skip connections to mitigate overfitting and improve anatomical structure modeling.

Despite the positive progress made by the aforementioned methods in various directions, several limitations remain. In terms of feature representation capability, traditional convolutional structures are relatively simple, limiting the ability of shallow features to characterize hippocampal boundaries and texture details. Regarding global context modeling, although the introduction of the vision transformer architecture enhances long-range dependency modeling, its integration with U-Net++ is relatively straightforward and fails to achieve effective multi-scale information interaction across spatial and channel dimensions. As for frequency-domain structural modeling, existing methods generally overlook periodic textures and low-frequency deformations in medical images, making it difficult to explicitly capture long-period structures in the frequency domain, thus limiting the modeling of hippocampal atrophy trends.

To address these issues, this paper proposes FED-UNet++, which enhances boundary feature representation in shallow layers through the FRBlock for boundary reconstruction. The EAP module is introduced to achieve compact multi-scale context modeling and reinforce deep semantic perception. Furthermore, the DFCN module is integrated to perform multi-scale spectral decomposition and adaptive frequency modulation, significantly improving the model’s ability to perceive blurred boundaries and low-frequency structures. While enhancing segmentation performance, this design keeps additional computational overhead within clinically acceptable limits, striking a balance between accuracy and practicality.

## 3. Materials and Methods

### 3.1. Architecture of the Proposed Method

This study proposes an improved U-Net++ architecture—FED-UNet++—aimed at enhancing the segmentation accuracy of the hippocampus in the auxiliary diagnosis of Alzheimer’s disease. Although U-Net++ enhances multi-scale semantic feature fusion through densely connected skip pathways, it still exhibits significant limitations in shallow feature extraction, deep contextual modeling, and global structural restoration. To address these challenges, FED-UNet++ introduces task-specific structural enhancement modules at different stages of the network—encoding, fusion, and decoding—forming a comprehensive optimization strategy characterized by “stage-wise deployment, structural complementarity, and collaborative enhancement.” The specific design includes:

Residual feature reconstruction module (FRBlock): Due to the simple convolutional structure in the original U-Net++ model, its shallow feature extraction is suboptimal for small-volume and boundary-blurred objects like the hippocampus. To address this issue, we propose the residual feature reconstruction module (FRBlock) to replace the original convolutional module. This module combines residual connections with feature enhancement mechanisms, employing a series of 1 × 1 and 3 × 3 convolutions to achieve feature compression and nonlinear reconstruction, thereby enhancing the representation of edge and texture details while preserving spatial resolution. The introduction of residual connections effectively alleviates the vanishing gradient problem and improves the stability of feature transmission and training robustness. In addition, an intermediate feature concatenation mechanism integrates low-dimensional semantic and high-dimensional structural features, strengthening the shallow layer’s ability to represent spatial boundaries and texture details. This design is particularly suitable for small-volume, low-contrast, and blurry-boundary structures such as the hippocampus.

Efficient attention pyramid module (EAP): To address the lack of multi-scale contextual modeling in the deep semantic encoding stage of U-Net++, which makes it difficult to accurately capture structural information in hippocampal regions with blurred boundaries and variable morphology, this study introduces EAP as a structural enhancement component at the end of the encoder. This module employs multi-scale atrous convolutions to obtain contextual information under different receptive fields. In addition, a direction-aware attention mechanism is incorporated to establish long-range dependencies in the spatial dimension, explicitly focusing on structurally critical regions. The synergy between multi-scale fusion and spatial attention equips the model with enhanced global perception and boundary localization capabilities. As a result, segmentation accuracy and structural consistency are effectively improved.

Dynamic frequency-domain context modeling module (DFCN): Although the U-Net++ architecture demonstrates strong local feature modeling capabilities, its decoder primarily relies on spatial convolution operations to integrate contextual information, making it difficult to effectively model long-range dependencies and fine-grained structures. To address this limitation, the DFCN module is deployed at the end of the decoder. This module leverages Fourier transform to capture low-frequency structures across regions as well as high-frequency details. A learnable frequency modulation mechanism is introduced to achieve adaptive feature reconstruction, effectively enhancing the model’s structural modeling and localization accuracy for targets with variable morphology and blurred boundaries, such as the hippocampus. The fusion of frequency-domain features and spatial-channel information further strengthens local responses, enabling unified representation of global consistency and local discriminability. This enhances the model’s contextual modeling capability for small-volume, boundary-blurred structures like the hippocampus.

**Figure 1:**
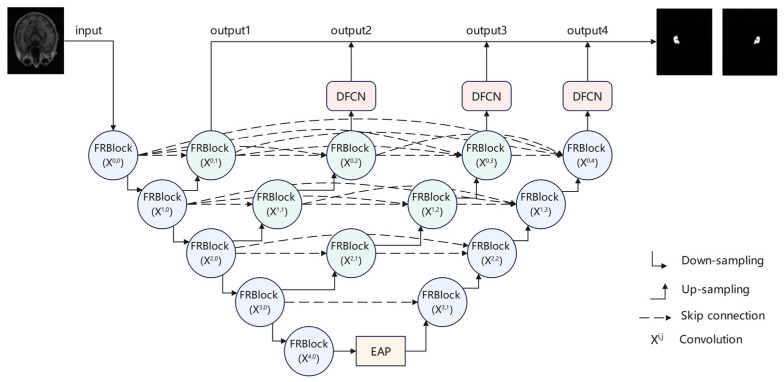
The architecture of the proposed FED-U-Net++.

The three modules are respectively applied to different structural stages—encoding, fusion, and decoding—forming a collaborative mechanism of targeted structural enhancement and functional complementarity. This enables the FED-UNet++ model to achieve a system-level optimization that surpasses conventional block-wise stacking in overall performance. The architecture of the proposed FED-UNet++ is illustrated in Figure 1.

### 3.2. Residual Feature Reconstruction Block (FRBlock)

To further enhance the feature extraction capability for hippocampal structures, this study improves upon the standard convolution modules in the original UNet++ architecture by proposing a novel residual feature reconstruction block (FRBlock). This module retains the residual connection structure while adopting a serial bottleneck design combined with a feature reconstruction fusion mechanism, aiming to increase feature representation depth and nonlinear modeling capability. By introducing a continuous combination of 1 × 1 and 3 × 3 convolutions for high-dimensional feature reconstruction, the module strengthens intermediate semantic modeling and significantly improves the model’s ability to capture complex textures and boundary information.

Specifically, the FRBlock first performs feature extraction using a standard 3 × 3 convolution when the input feature map is as follows: $X\in R^{B\times C_{in}\times H\times W}$. First, a standard 3 × 3 convolution is applied to extract low-level features, followed by batch normalization and the ELU activation function for feature normalization and nonlinear activation, thereby enhancing the nonlinear representation capability of the network. Compared with the ReLU activation function, ELU provides better gradient propagation in the negative value domain. The mathematical expression of this process is given as follows:

(1) $$F_{1}=ELU\left(BN\left({Conv}_{3\times 3}\left(X\right)\right)\right)$$

(2) $$F_{1}\epsilon R^{B\times C_{mid}\times H\times W}$$

The initially extracted features are then further reconstructed, starting with a 1 × 1 convolution to reduce the feature dimensionality, which decreases the number of parameters while retaining essential information, followed by a 3 × 3 convolution for further feature extraction, enhancing the feature representation capability. This sequence of operations enables feature enhancement and nonlinear mapping, and the combination of sequential 1 × 1 and 3 × 3 convolutions captures richer contextual information.

The mathematical expression of this process is given as follows:

(3) $$F_{2}=ELU\left({Conv}_{3\times 3}(ELU({Conv}_{1\times 1}(F_{1}))\right))$$

(4) $$F_{2}\epsilon R^{B\times C_{mid}\times H\times W}$$

The feature map F1 extracted in the first step and the reconstructed feature map F2 from the second step share the same spatial dimensions (H × W) and the same number of channels $C_{mid}$, F1 and F2 are then concatenated along the channel dimension, resulting in a channel number of ${2C}_{mid}$. This concatenation allows the model to exploit the relationship between low-level features and high-dimensional reconstructed features. A 1 × 1 convolution is subsequently applied to the concatenated feature map to perform feature fusion and map it to the output channel dimension $C_{out}$. Batch normalization and the ELU activation function are applied to ensure the nonlinear representation capability and numerical stability of the network. This process can be mathematically expressed as follows:

(5) $$F_{fused}=ELU(BN({Conv}_{1\times 1}({[F}_{1},F_{2}])))$$

(6) $$F_{fused}\in R^{B\times C_{out}\times H\times W}$$

A residual connection is introduced at the final stage of the FRBlock module. If the number of input channels does not match the number of output channels ($C_{in}\neq C_{out}$), a 1 × 1 convolution is used for dimensional alignment. The input feature map, once adjusted for dimensional consistency, is subsequently combined with the output from the previous processing steps. Finally, the result is processed by the ELU activation function. The residual connection helps maintain network stability, ensuring more effective gradient flow during backpropagation and mitigating the problem of gradient vanishing in deep networks. The mathematical formulation of this process is as follows:

(7) $$Y=ELU(F_{fused}+Shortcut(X))$$

(8) $$Output\in R^{B\times C_{out}\times H\times W}$$

**Figure 2:**
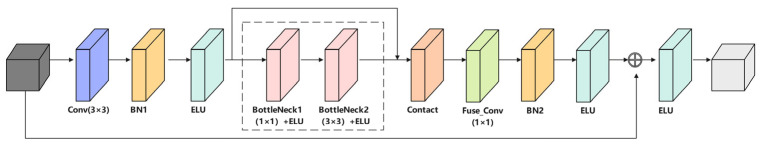
The architecture of the proposed Residual Feature Reconstruction Block (FRBlock). The dashed box highlights the serial bottleneck structure (1 × 1 and 3 × 3 convolutions) for feature reconstruction, while the arrows indicate the directional flow of feature maps within the block.

The residual feature reconstruction block (FRBlock) is illustrated in Figure 2.

Unlike the classic ResNet residual bottleneck structure, which typically adopts a 1 × 1 → 3 × 3 → 1 × 1 convolutional sequence with residual addition at the output stage, the proposed FRBlock introduces a serial feature reconstruction mechanism specifically designed for medical image segmentation tasks. In this design, the input features are first processed by a 3 × 3 convolution to extract local spatial context, followed by a sequential path of 1 × 1 and 3 × 3 convolutions for further feature refinement. The original features and the reconstructed features are concatenated along the channel dimension and fused via a 1 × 1 convolution.

Compared to the additive fusion at the output stage in ResNet, this channel concatenation-based fusion retains richer spatial and semantic information. Meanwhile, FRBlock preserves the residual connection structure by adding the fused output to the original input (or its channel-aligned version), which helps stabilize gradient propagation and accelerate model convergence.

In summary, although both structures use similar convolutional operators (1 × 1 and 3 × 3), FRBlock fundamentally differs from the ResNet bottleneck in structure through its serial enhancement design, channel fusion strategy, and segmentation-oriented architecture, making it more suitable for dense prediction tasks such as hippocampus segmentation.

### 3.3. Efficient Attention Pyramid Module (EAP)

**Figure 3:**
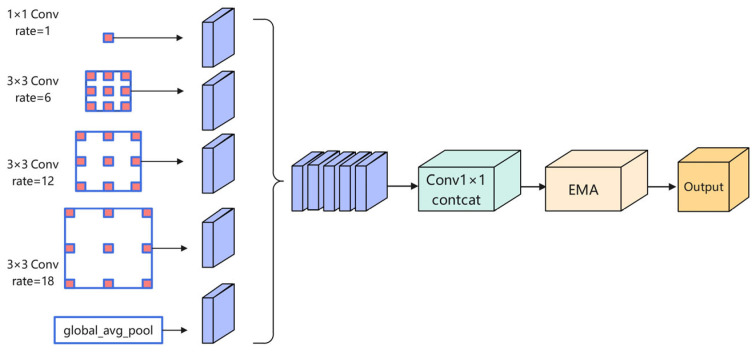
Efficient attention pyramid (EAP) module.

As shown in Figure 3, to strengthen multi-scale feature representation and refine the model’s attention to salient regions, thereby strengthening the spatial perception of deep features to brain structures, this paper proposes an efficient attention pyramid (EAP) module at the deepest layer of UNet++ (i.e., conv4_0 at the encoder’s end).

**Figure 4:**
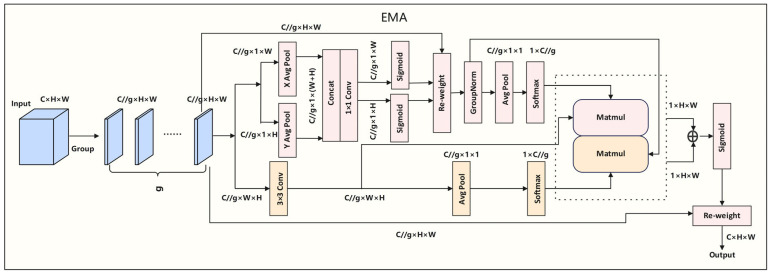
Efficient multi-scale attention (EMA) module. The dashed box highlights the attention modeling unit, where horizontal and vertical contextual dependencies are captured through pooling and matrix multiplication (MatMul). The arrows indicate the flow of feature information between different operations.

Based on the classical ASPP structure, this module integrates the efficient multi-scale attention (EMA) mechanism (Figure 4) to capture long-range contextual relationships in both horizontal and vertical dimensions, thereby improving sensitivity to critical anatomical areas like the hippocampus. This design integrates multi-scale contextual information with direction-sensitive attention mechanisms, offering stronger structural adaptability and representation capability compared to conventional serial approaches.

Specifically, the SAP module first employs an atrous spatial pyramid pooling (ASPP) structure, utilizing multiple parallel convolution branches with different dilation rates to extract contextual information from various receptive fields, thereby enhancing multi-scale feature representation. Feature maps from all branches are first concatenated across the channel axis, then integrated via a 1 × 1 convolution to produce a representation with enhanced multi-scale contextual features.

On this basis, an efficient multi-scale attention module (EMA) is introduced to build directional spatial dependencies. This component first applies average pooling along the horizontal and vertical directions to extract directional contextual features; then it uses a 1 × 1 convolution to generate initial attention maps and performs matrix multiplication (MatMul) to establish cross-dimensional spatial dependencies, achieving joint modeling of spatial and channel dimensions. The resulting attention weights are used to perform weighted reconstruction on the input feature map, highlighting key region features while suppressing background noise, thus significantly enhancing the expression quality of hippocampal boundaries and the segmentation accuracy.

Specifically, the input feature map $X\in R^{B\times C_{in}\times H\times W}$ is first processed by atrous convolutions with different dilation rates r∈{1,6,12,18} to extract multi-scale contextual information, resulting in four parallel branches:

(9) $$F_{i}={Conv}_{3\times 3}^{r_{i}}\left(X\right),i\in \{1,2,3,4\}$$

To obtain multi-scale contextual information, the EAP module introduces five parallel branches, including a 1 × 1 convolution branch F0, three 3 × 3 atrous convolution branches $F_{1}$~$F_{3}$ with different dilation rates, and a global average pooling branch $F_{4}$. The dilation rate configuration [1,6,12,18] is adopted as the default setting, which is widely used in multi-scale receptive field modeling tasks such as DeepLabv3+.This configuration provides rich contextual information without introducing significant computational overhead. Experimental results show that this configuration performs consistently well for the task at hand, and therefore it is retained in this study. The outputs of the five branches are concatenated along the channel dimension to obtain the fused feature representation:

(10) $$F_{cat}=Concat(F_{0},F_{1},F_{2},F_{3},F_{4})$$

To further enhance the representation capacity of the fused features, the EMA module is introduced for attention enhancement. The fused features are first subjected to channel compression:

(11) $$F_{r}={Conv}_{1\times 1}(F_{cat})$$

Subsequently, attention modeling is performed on $F_{r}$ by combining horizontal pooling $P_{h}$ and vertical pooling $P_{w}$ to capture contextual information along both directions:

(12) $$F_{h}=P_{h}(F_{r})$$

(13) $$F_{w}=P_{w}(F_{r})$$

Subsequently, the information from both directions is fused and subjected to attention mapping:

(14) $$F_{aat}=\sigma ({Conv}_{1\times 1}(Contact(F_{h},F_{w})))$$

where σ denotes the sigmoid activation function, which is used to generate the spatial attention weights. The final output is derived by performing an element-wise multiplication between the input features and the computed attention map:

(15) $$Y=F_{r}\cdot F_{aat}+F_{r}$$

The EAP module captures multi-scale contextual information through atrous convolutions and enhances spatial focus via an efficient lightweight attention mechanism, effectively improving the model’s perception and structural modeling capability for hippocampal boundary regions.

To further clarify the distinctions between the proposed EAP module and existing attention-fusion mechanisms such as CBAM-ASPP and pyramid attention, we present the following comparisons:

First, CBAM-ASPP enhances features by sequentially applying channel and spatial attention modules after ASPP, acting as a post-processing refinement. In contrast, our EAP module directly integrates attention enhancement into the pyramid fusion process by embedding the EMA mechanism within the multi-branch ASPP output. This unified design facilitates more compact and efficient contextual representation learning during feature aggregation.

Second, unlike the top-down, coarse-to-fine attention guidance adopted in pyramid attention, EAP combines a parallel multi-scale feature extraction structure (via ASPP) with a serial, direction-aware attention mechanism (via EMA). Specifically, EMA performs directional pooling along horizontal and vertical axes, followed by matrix-based dependency modeling, enabling spatially fine-grained attention modulation.

These design differences allow EAP to more effectively capture subtle anatomical boundaries and contextual variations, particularly in complex medical structures such as the hippocampus. As demonstrated in our experiments, the EAP module leads to superior accuracy and clearer boundary delineation compared with conventional attention-fusion mechanisms in medical image segmentation.

### 3.4. Dynamic Frequency Context Network (DFCN)

**Figure 5:**
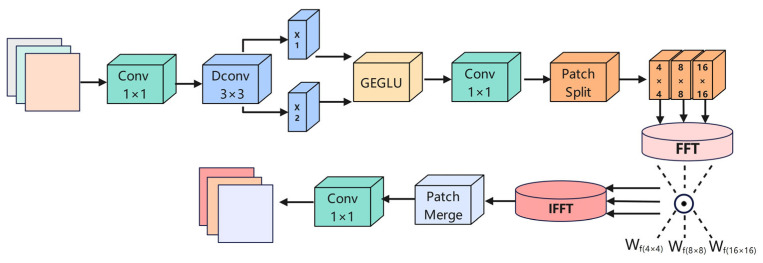
The dynamic frequency context network (DFCN) module.

Although U-Net++ provides a solid structural foundation for multi-scale feature fusion, its decoder mainly relies on local convolution operations, lacking effective modeling of long-range dependencies and periodic structures. In hippocampus segmentation, anatomical structures are typically small and curved, with blurred boundaries and low contrast in MRI images. Frequency-domain modeling is particularly effective for such tasks, as it can simultaneously capture low-frequency global contextual information (for maintaining shape consistency) and high-frequency local boundary cues (for accurate boundary localization). To address this limitation, inspired by the EDFFN module, we propose a dynamic frequency context network (DFCN) (Figure 5) and integrate it into several deep stages of the decoder to enhance global awareness and structural representation of high-level semantic features. The proposed DFCN module strengthens boundary clarity and preserves fine-grained structural morphology through multi-scale spectral decomposition and adaptive frequency modulation. This joint modeling approach is particularly suitable for the hippocampal region, offering clear advantages when spatial convolutions alone struggle to detect subtle changes in atrophic or compressed tissue.

The DFCN module centers on frequency-domain modeling. It first applies depthwise separable convolution to extract spatial structure information from the input feature map, which is then divided into patches of multiple scales (e.g., 4 × 4, 8 × 8, 16 × 16), followed by independent 2D Fourier transforms (FFT) at each scale. In the frequency domain, the feature maps are element-wise multiplied with learnable frequency modulation weights, enabling dynamic amplitude modulation and response reconstruction. The results are then transformed back to the spatial domain via inverse FFT (IFFT), followed by channel-wise fusion of multi-scale frequency features and integration using a 1 × 1 convolution. This frequency-response-based modeling strategy enables DFCN to effectively capture long-range semantic relationships and fine-grained structural variations (e.g., hippocampal boundaries and tail regions), substantially compensating for the decoder’s limited global context awareness due to its reliance on local convolution. Given that shallow features primarily contain low-level textures, where frequency-domain enhancement is less effective, the DFCN module is deployed after the FRBlock in deeper decoder substructures (e.g., conv0_2 to conv0_4), operating on the fused multi-scale semantic feature maps to fully leverage the advantages of frequency modeling.

Experimental findings indicate that the DFCN module enhances the model’s capability to accurately identify intricate small-scale structures, including the hippocampus, in high-resolution MRI scans, while maintaining a controllable parameter overhead, thus confirming its reliability and applicability in real-world medical image segmentation scenarios.

The proposed DFCN module structurally integrates three components: depthwise separable convolution, multi-scale frequency-domain dynamic filtering, and projection-based fusion. It begins with channel expansion and depthwise convolution to extract local features. Given an input feature map $X\in R^{B\times C\times W\times H}$, where B is the batch size and C is the number of channels, a 1 × 1 convolution is applied to double the channel dimension:

(16) $$X_{ext}={Conv}_{1\times 1}(X)\in R^{B\times 2C\times W\times H}$$

The expanded feature map is then split along the channel dimension into two separate branches:

(17) X_1,X_2=Split(X_ext,dim=1)

Each branch independently extracts local features through depthwise separable convolution, followed by a gated feedforward mechanism to enhance feature representations and integrate nonlinear selection capabilities:

(18) $$X_{local}=GEGLU(X_{1}){⊙X}_{2}$$

Here, $X_{1}$ and $X_{2}$ represent the two branches obtained from channel splitting, and ⊗ denotes element-wise multiplication used to enhance the response of salient channels. This step strengthens the model’s ability to capture hippocampal atrophy boundaries and improves the accuracy of boundary segmentation.

The process then enters the frequency-domain dynamic filtering stage. To better balance local and global information, the original fixed-size P × P patch division strategy is replaced with a pyramidal multi-scale partitioning scheme. Specifically, the feature map $X_{local}\in R^{B\times C\times H\times W}$ is divided into three groups of non-overlapping patches with sizes of P_1_ = 4, P_2_ = 8, and P_3_ = 16, respectively. To enhance the multi-scale adaptability of frequency-domain modeling, the DFCN module adopts a pyramid-style patch partitioning strategy, where the feature map is divided into non-overlapping patches of sizes 4 × 4, 8 × 8, and 16 × 16. This design is inspired by the widely used multi-scale frequency decomposition methods in the fields of image deblurring and frequency-domain reconstruction, which have been proven effective and efficient in modeling structures at different frequency levels. By capturing global contextual structures at low-frequency scales (e.g., 16 × 16) and enhancing boundary detail awareness at high-frequency scales (e.g., 4 × 4), this configuration improves segmentation accuracy while keeping computational complexity under control. Preliminary experiments demonstrate that this three-scale combination exhibits strong stability and generalization performance in hippocampus segmentation tasks, with particularly notable improvements in boundary modeling quality. Therefore, this setting is adopted as the default parameter configuration for the DFCN module in this study:

(19) $$X^{\left(s\right)}={\left\{X_{k}^{\left(s\right)}\right\}}_{k=1}^{N_{s}},s\in \{1,2,3\}$$

where s denotes the scale index and ${\;N}_{s}$ is the number of patches at the corresponding scale. Each patch at every scale is independently transformed using a 2D real-valued fast Fourier transform (FFT):

(20) $$F_{k}^{\left(s\right)}=FFT2(X_{k}^{\left(s\right)})\in C^{B\times C\times P_{s}\times (P_{s}/2+1)}$$

Subsequently, frequency domain modulation is performed using the learnable amplitude weights $W_{f}^{\left(s\right)}$ associated with each scale:

(21) $${\tilde{F}}_{k}^{\left(s\right)}=F_{k}^{\left(s\right)}⊙W_{f}^{\left(s\right)}$$

The spatial domain patches are restored via inverse Fourier transform (IFFT):

(22) $${\tilde{X}}_{k}^{\left(s\right)}=IFFT2({\tilde{F}}_{k}^{\left(s\right)})$$

After all patches at different scales complete the above processes, they are concatenated along the channel dimension via the PatchMerge operation:

(23) $$X_{frcq}=Concat({\tilde{X}}^{\left(1\right)},{\tilde{X}}^{\left(2\right)},{\tilde{X}}^{\left(3\right)})\in R^{B\times 3C\times H\times W}$$

The scale-specific frequency-domain representation effectively integrates structural information from different receptive fields, significantly enhancing the model’s ability to jointly model low-frequency large-scale morphology and high-frequency edge details.

Finally comes the projection and fusion, where the enhanced feature maps are projected back to the original channel dimension to complete the feature fusion, using a 1 × 1 convolution to compress the number of channels:

(24) $$Y={Conv}_{1\times 1}(X_{frcq})\in R^{B\times C\times H\times W}$$

The feature map Y integrates boundary awareness from the local enhancement path and global structural modeling from the multi-scale frequency-domain path, enabling multi-scale joint modeling of brain structures. It is especially effective in identifying mild atrophic patterns within key brain regions, including the hippocampus, on MRI scans of Alzheimer’s disease patients.

Compared with existing frequency-domain methods such as Fourier attention and FFCNet, the proposed DFCN module offers the following advantages:

First, Awhile Fourier attention performs spectral modeling on fixed-size patches, DFCN introduces multi-scale frequency partitioning combined with learnable frequency modulation, enabling adaptive capture of semantic variations across different structural scales.

Second, FFCNet adopts a local-global fusion design but does not explicitly incorporate frequency-domain transformations. In contrast, DFCN utilizes explicit FFT/IFFT operations along with frequency filtering, significantly improving its responsiveness to structural boundaries.

Finally, DFCN integrates depthwise separable convolution, gated enhancement, and dynamic frequency modeling in a unified lightweight framework, achieving a balance between computational efficiency and representational capacity. These design features yield notable advantages in boundary refinement and small-structure segmentation tasks such as the hippocampus.

## 4. Experiments

This section first introduces the datasets used for hippocampal segmentation in Alzheimer’s disease, along with the associated evaluation metrics and experimental settings. Next, the proposed model is systematically compared with several state-of-the-art segmentation methods. Finally, ablation studies are conducted to evaluate the individual contributions of the components integrated into the FED-UNet++ framework.

### 4.1. Datasets

In this study, we employed two publicly available hippocampus MRI segmentation datasets to train and evaluate the proposed model. The details are as follows:

Kaggle hippocampus segmentation dataset: This dataset originates from a publicly released dataset on the Kaggle platform (published by Malekzadeh in 2019) [18] and is specifically designed for the automatic segmentation of hippocampal structures in MRI images of Alzheimer’s disease patients. The dataset derives from neuroimaging data provided by the Alzheimer’s Disease Neuroimaging Initiative (ADNI) [36], with manual annotations of the left and right hippocampi following preprocessing, offering high clinical reference value. The training set comprises T1-weighted MRI scans from 100 patients with Alzheimer’s disease, while the test set includes scans from 35 patients. We selected images with valid left hippocampus masks for this study, resulting in a total of 1003 images in the training set and 252 images in the test set.

It is worth noting that the ADNI database includes patient data spanning multiple age groups (e.g., from 60 to over 90 years old) and various stages of Alzheimer’s disease progression, including mild cognitive impairment (MCI), early-stage AD, and late-stage AD. Since the dataset published on Kaggle is constructed based on ADNI data, it contains individuals with diverse age ranges and disease severities. This sample diversity helps improve the model’s generalization ability across different pathological conditions, thereby enhancing its practicality and robustness.

Task004_Hippocampus: This dataset is derived from the publicly available Task004_Hippocampus dataset provided by the Medical Segmentation Decathlon (MSD) [19]. The dataset focuses on accurate hippocampus segmentation and is widely used in studies and auxiliary diagnosis of neurodegenerative diseases such as Alzheimer’s disease. It contains a total of 260 MRI scans, each consisting of 3D T1-weighted MRI data along with precise annotations. To accommodate the 2D segmentation model used in this study, the 3D MRI data were axially sliced, and slices containing valid hippocampal structure masks were selected. The dataset was partitioned into 1052 training slices and 263 testing slices using an 8:2 split ratio.

To prevent data leakage between the training and testing phases, a subject-exclusive splitting strategy is adopted for both datasets in this study. That is, all images in the training and testing sets come from different individuals. As a result, no brain slices from the same subject appear in both the training and testing sets, thereby ensuring the validity, fairness, and generalizability of the model evaluation results.

### 4.2. Experimental Settings and Parameters

The input data were uniformly resized to 256 × 256 before being fed into the model, with random flipping, rotation, and HSV augmentation applied. These operations effectively augmented the training set and improved the model’s generalization and robustness. Initially, the SGD optimizer was used, but due to its slow convergence, we eventually switched to the more effective Adam optimizer. A weight decay of 1 × 10^−4^ was employed, and the training began with a learning rate of 1 × 10^−3^, adjusted using a cosine annealing scheduler. This scheduler gradually decayed the learning rate to a minimum of 1 × 10^−5^ during training. Such a configuration helped improve convergence stability and prevented overfitting. FED-UNet++ was trained for 300 epochs with a batch size of 16. The FED-UNet++ model was implemented using PyTorch1.8.1 and trained on a workstation equipped with an RTX 3090 GPU under identical settings.

### 4.3. Evaluation MetricsS

Our experiments employed five widely used segmentation metrics: IoU, Dice, recall, precision, accuracy, and HD95. The intersection over union (IoU) quantifies the agreement between predicted and actual hippocampal regions, where larger values reflect improved segmentation accuracy, as specified in Equation (25). Dice evaluates the similarity between the predicted results and the ground truth, as shown in Equation (26). Recall quantifies how many of the actual foreground pixels are correctly detected by the model, reflecting the rate of missed detections; higher values indicate fewer omissions, as shown in Equation (27). Precision measures the proportion of predicted foreground pixels that are truly foreground. This metric reflects the model’s false positive rate; higher values indicate fewer false segmentations, as defined in Equation (28). Accuracy denotes the proportion of correctly predicted pixels, including both foreground and background, thus reflecting the overall segmentation performance of the model, as shown in Equation (29). As shown in Equation (30), HD95 measures the maximum error distribution between the predicted segmentation boundary and the ground truth boundary. Specifically, it refers to the 95th percentile value in the distance distribution from the predicted boundary to the ground truth boundary. Compared to the traditional Hausdorff distance (which takes the maximum distance), HD95 is more robust and reduces the influence of extreme outliers on the result.

In our experiments, we employ a tailored loss function that integrates binary cross-entropy (BCE) and Dice losses to mitigate class imbalance and improve the model’s focus on target region learning. The final loss function is a weighted sum of BCE and Dice losses. Each component is assigned a weight of 0.5, indicating equal importance during loss computation. The rationale behind this setting is to balance pixel-level accuracy and regional structural consistency while maintaining numerical stability. To verify the robustness of the equal weighting scheme between BCE and Dice, this study conducted a sensitivity analysis on the FED-UNet++ model under different loss weight combinations (BCE:Dice = 0.7:0.3, 0.5:0.5, 0.3:0.7). As shown in Table 1, evaluation metrics across different settings showed minimal variation, indicating that the loss function maintains stable performance even under class imbalance conditions. Among them, the equal weighting configuration (0.5:0.5) between BCE and Dice achieved the best performance on multiple metrics. Therefore, this configuration was ultimately selected as the default setting to balance segmentation contour accuracy and regional overlap precision.

(25) $$IoU=\frac{TP}{TP+FP+FN}$$

(26) $$Dice=\frac{2TP}{2TP+FP+FN}$$

(27) $$Recall=\frac{TP}{TP+FN}$$

(28) $$Precision=\frac{TP}{TP+FP}$$

(29) $$Accuracy=\frac{TP+TN}{TP+TN+FP+FN}$$

(30) $${HD}_{95}(A,B)=max\left\{{percentile}_{95}\left(\underset{b\in B}{\min}\left||a-b|\right||a\in A\right),{percentile}_{95}\left(\underset{a\in A}{\min}\left||b-a|\right||b\in B\right)\right\}$$

In this context, TP refers to correctly identified foreground pixels, FP to background pixels misclassified as foreground, FN to actual foreground pixels not detected, and TN to background pixels accurately classified. A denotes the set of predicted boundary points, B represents the set of ground truth boundary points, ||a−b|| refers to the Euclidean distance, and ${percentile}_{95}$ indicates the 95th percentile value of the distance distribution.

### 4.4. Results

To comprehensively validate the effectiveness of the proposed FED-UNet++ model, this section presents extensive experiments and analyses. Specifically, we include comparisons with state-of-the-art models (Section 4.4.1), ablation studies of the key modules (Section 4.4.2), and detailed performance evaluations on two benchmark hippocampus MRI datasets (Section 4.4.3 and Section 4.4.4).

#### 4.4.1. Results and Analysis

**Figure 6:**
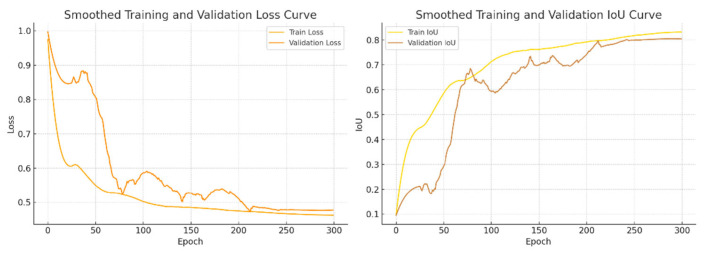
Training and validation curves of the FED-UNet++ model.

To evaluate the training stability and convergence behavior of the proposed model, Figure 6 presents the loss and IoU curves during both the training and validation phases. As shown, the training loss continuously decreases throughout the training process and stabilizes after approximately 200 epochs. The IoU on the validation set also exhibits an overall upward trend. Although the validation curve shows some fluctuations, this is primarily attributed to the anatomical characteristics of the hippocampus—namely, its small volume and indistinct boundaries—which make it susceptible to image heterogeneity and inter-subject variability in the validation set. These factors may cause slight variations in prediction performance across different images. Nevertheless, the overall trend remains stable, and the final metrics converge well, confirming the model’s good convergence and generalization capability.

To demonstrate the superiority of our model, we compared it with several advanced and classical segmentation networks, including U-Net, U-Net++, SwinUNet, PSPNet, and DeepLabv3+, which are widely regarded as baselines for medical image segmentation. All models were trained under the same settings using the first Kaggle dataset. Table 1 reports the performance in terms of IoU, Dice, precision, recall, and accuracy.

By comparing the results in Table 2, it can be concluded that our proposed model outperforms other baseline models on the Kaggle dataset (left hippocampus). Specifically, the IoU reaches 0.7495, which is 4.44% higher than the original U-Net++, highlighting the superior robustness and accuracy of our model in small-structure segmentation tasks.

#### 4.4.2. Ablation Study of Key Modules

A set of ablation studies was performed to assess the individual contribution of core components, including the residual feature reconstruction block (FRBlock), the efficient attention pyramid (EAP), and the dynamic frequency context network (DFCN). Table 3 presents the performance changes observed when progressively integrating each module into the baseline U-Net++ model. To ensure the statistical reliability of the improvement in the Dice metric, each experimental setting was trained and evaluated five times using different random seeds (41, 77, 123, 2024, 888), and the mean and standard deviation of the Dice scores were calculated. The experimental results demonstrate that each component contributes positively to the overall performance improvement, validating their practical utility and effectiveness in the hippocampus segmentation task.

In the following, FR+ denotes the integration of the feature reconstruction block (FRBlock) into the network, EA+ indicates the inclusion of the efficient attention pyramid (EAP) module, and DF+ represents the incorporation of the dynamic frequency context network (DFCN) module.

To evaluate the effectiveness of FED-UNet++ in hippocampus segmentation, we conducted ablation studies on the publicly available Kaggle dataset, using U-Net++ as the baseline. Specifically, to assess the contribution of the FRBlock module, we replaced the standard convolutional blocks in U-Net++ with FRBlock while keeping other components unchanged.

In the visualization of segmentation results (Table 4), the output images were cropped around the left hippocampal region to facilitate the observation of structural morphology and boundary characteristics, given the relatively small size of the hippocampus within brain images. It should be noted that this cropping was performed solely for visualization purposes and does not affect the actual output dimensions of the segmentation results.

Specifically, the IoU increased from 70.51% to 72.10%, and the Dice coefficient improved from 82.36% to 83.41%, recall rose from 80.80% to 82.10%, and precision increased from 84.58% to 85.21%. The overall accuracy remained at 99.88%, slightly higher than that of the baseline model (99.87%).

These performance gains are primarily attributed to the enhanced feature extraction capabilities of the FRBlock. By incorporating residual connections and more expressive convolutional operations, this module enables the shallow encoder layers to better preserve low-level spatial details while improving the efficiency of gradient propagation. This architectural enhancement increases the model’s sensitivity to subtle hippocampal boundaries, which is particularly important for volumetric analysis in the early diagnosis of Alzheimer’s disease.

Furthermore, when the FRBlock is integrated with other modules such as EAP and DFCN, the overall network performance is further enhanced. Specifically, the combination of all three modules leads to a significant improvement in segmentation accuracy, achieving an IoU of 74.95% and a Dice coefficient of 85.42%. These results indicate that the FRBlock effectively complements both multi-scale contextual modeling and frequency-domain representation, serving as a foundational component for improving hippocampal segmentation performance. Despite the substantial performance gains, the inclusion of the FRBlock introduces only minimal computational overhead, making it a cost-effective architectural enhancement for medical image segmentation tasks.

To validate the effectiveness of the proposed EAP module in enhancing hippocampus segmentation, we integrated it into the final stage of the U-Net++ encoder and performed ablation studies. Compared with the baseline model, the introduction of EAP resulted in notable improvements across multiple evaluation metrics.

Specifically, the IoU increased from 70.51% to 72.29%, the Dice coefficient improved from 82.36% to 83.62%, while recall and precision rose from 80.80% and 84.58% to 84.37% and 83.40%, respectively. The overall accuracy remained consistently high at 99.88%. These performance gains are primarily attributed to the EAP module’s capability to capture multi-scale contextual information and enhance global structural perception. Unlike conventional ASPP, EAP incorporates the EMA attention mechanism, which facilitates semantic correlation modeling across spatial locations through cross-position attention. This design offers significant advantages in segmenting anatomically complex and poorly defined regions such as the hippocampus.

Moreover, the EAP module facilitates enhanced semantic fusion across multiple scales and strengthens the network’s capacity to model long-range dependencies and AD-specific atrophy patterns. When further combined with the FRBlock or EDFFN module, the overall network performance is further improved, demonstrating the strong complementarity of EAP within the proposed architecture. Overall, EAP serves as an efficient and lightweight encoder enhancement module that substantially improves multi-scale representation capabilities while incurring minimal additional computational cost.

To evaluate the effectiveness of the proposed dynamic frequency context network (DFCN) module, it was integrated into the deep decoder layers of the U-Net++ architecture, and ablation studies were conducted on the Kaggle hippocampus segmentation dataset. Compared with the baseline model, the introduction of DFCN consistently improved boundary delineation and enhanced the recognition of small-scale anatomical structures.

Specifically, the IoU increased from 70.51% to 72.45%, the Dice coefficient rose from 82.36% to 83.66%, recall improved from 80.80% to 83.51%, and precision increased from 84.58% to 84.47%, while the overall accuracy remained at 99.88%. These performance improvements can be attributed to the effective synergy of the DFCN module in enhancing spatial detail and modeling frequency-domain contextual features. On one hand, the module leverages depthwise separable convolutions to efficiently extract local structures and edge details. On the other hand, it partitions feature maps into multiple non-overlapping patches, applies fast Fourier transform (FFT) to each patch, and performs dynamic frequency-domain modulation using learnable amplitude weights. This enables the network to capture periodic patterns and long-range dependencies that conventional convolutions typically fail to model.

In addition, as evidenced by the ablation results in Table 3, integrating the DFCN module into the baseline model leads to significant improvements in key performance metrics such as IoU and Dice. The visualized segmentation results further corroborate this enhancement: in the final experimental group, the model incorporating DFCN (e.g., the second image within the red bounding box) demonstrates improved preservation of hippocampal continuity. These findings confirm the effectiveness and practical value of frequency-domain modeling in hippocampal segmentation. Notably, the specific architectural design of the DFCN module renders it particularly effective in capturing the structural and contextual complexities of the hippocampus, such as its curved morphology and indistinct boundaries.

By bridging the frequency and spatial domains, the DFCN module facilitates the integration of global and local semantic information, thereby enhancing the model’s capability to interpret complex anatomical structures. In Alzheimer’s-related medical imaging, such fusion is particularly crucial, as it enables more sensitive detection of boundary blurring and morphological changes associated with mild hippocampal atrophy, thereby assisting in the early diagnosis of Alzheimer’s disease. When combined with other modules such as FRBlock and EAP, the model achieved an IoU of 74.95% and a Dice coefficient of 85.42%, further demonstrating the effectiveness and complementary value of the DFCN module within complex segmentation architectures.

To further validate the effectiveness and interpretability of the proposed modules, visual analyses were performed on the attention activation maps and frequency responses of the EAP and DFCN modules, respectively. Table 5 presents representative examples demonstrating the modules’ ability to focus on hippocampal boundary regions. As illustrated, the attention heatmap generated by the EAP module shows pronounced activation in the left hippocampal area, which closely aligns with the intended segmentation target. Despite minor deviations, the high-response regions largely coincide with the structural boundaries of the hippocampus, confirming the EAP module’s capability to highlight spatially salient areas. Furthermore, the frequency response visualizations of the DFCN module reveal significantly enhanced activation near hippocampal boundaries, indicating its effectiveness in modeling fine structural details in the frequency domain while suppressing irrelevant background noise. 

The above visualization results demonstrate that both the EAP and DFCN modules not only improve the model’s segmentation performance—reflected in higher IoU and Dice scores—but also enable effective focus on hippocampal boundaries at the feature level, thereby enhancing the reliability and interpretability of medical image segmentation.

As illustrated in Table 6, although the proposed FED-UNet++ model achieves excellent overall segmentation performance, certain challenges remain in cases involving blurred hippocampal boundaries and subtle atrophic changes. These failure cases highlight the need for further improvements in handling small anatomical structures and ambiguous boundary regions. Future work will focus on optimizing the model’s robustness and adaptability in such challenging scenarios.

#### 4.4.3. Robustness to Noise

To further evaluate the robustness of the proposed FED-UNet++ under challenging conditions, we introduced Gaussian noise (σ = 15) into the test set to simulate MRI artifacts. The visual comparison of segmentation results before and after noise interference is illustrated in Table 7, while the quantitative performance evaluation is reported in Table 8, although a moderate performance drop was observed, the model retained stable segmentation accuracy, with IoU and Dice scores decreasing by only 2.32% and 1.70%, respectively. This confirms the potential reliability of FED-UNet++ in real-world noisy clinical environments.

#### 4.4.4. Generalization Experiments

To assess the proposed model’s generalization capability and performance across different datasets, additional experiments were carried out using the Task04_Hippocampus public dataset. In this study, 3D brain MRI volumetric data were sliced along the axial plane into 2D images for hippocampus segmentation, based on the following considerations. First, compared to 3D networks, 2D convolutional networks are more lightweight in terms of parameter count and memory consumption, making them more suitable for deployment in resource-constrained clinical environments. Second, as a slender and small structure, the hippocampus exhibits relatively complete spatial feature expression in axial slices. A single frame can contain sufficient anatomical information to support accurate segmentation results.

We selected the baseline U-Net++ model and three key configurations for comparative analysis, including Baseline + FRBlock, Baseline + FRBlock + EAP, and the final complete FED-UNet++ model. To enhance the comparison, several representative segmentation models were also included, such as the classical U-Net, PSPNet, DeepLabv3+, and the transformer-based SwinUNet. By training and evaluating all models under a unified setting, we further confirmed the superior performance and strong generalization ability of the proposed model across diverse data scenarios.

Table 9 demonstrates that the proposed FED-UNet++ model exhibits strong generalization capabilities on the Task04_Hippocampus dataset, outperforming both classical and contemporary state-of-the-art segmentation methods. The baseline U-Net++ model achieved an IoU of 79.23% and a Dice coefficient of 88.22%. In comparison, the final FED-UNet++ model—which integrates the residual feature reconstruction block (FRBlock), efficient attention pyramid (EAP), and dynamic frequency context network (DFCN)—achieved the best overall performance across all evaluation metrics, with an IoU of 82.51%, Dice of 90.39%, recall of 89.86%, precision of 90.94%, and overall pixel accuracy of 98.65%.

Compared with traditional models such as U-Net, PSPNet, and DeepLabv3+, FED-UNet++ demonstrates superior capability in boundary detail preservation, semantic feature modeling, and adaptability to anatomical variability, underscoring its robustness and versatility. Notably, when compared with the baseline U-Net++, FED-UNet++ improves IoU by 3.28% and Dice by 2.17%, confirming its strong generalization capacity in cross-dataset segmentation tasks.

Furthermore, ablation experiments reveal that model performance consistently improves with the successive integration of FRBlock, EAP, and DFCN, thereby validating the individual effectiveness and synergistic contributions of each module. Collectively, these results highlight that FED-UNet++ constitutes an efficient, stable, and broadly applicable framework for medical image segmentation. As further illustrated in Table 10.

While we acknowledge that processing individual 2D slices may result in the loss of inter-slice contextual information, 2D models offer several practical advantages. Specifically, they enable more diverse data augmentation strategies during training and generally exhibit faster convergence. These benefits contribute to improved generalization performance. In future work, we plan to incorporate lightweight 3D modeling techniques to enhance the model’s capacity for capturing volumetric continuity while maintaining acceptable computational complexity.

#### 4.4.5. Model Efficiency Analysis

To further validate the resource efficiency of the proposed modules, we compared the computational cost of FRBlock, EAP, and EDFN with the original VGGBlock module in U-Net++ under the same channel settings. The evaluation includes metrics such as the number of parameters, FLOPs, and GPU memory usage. The results are presented in Table 11.

Although the proposed FRBlock, EAP, and DFCN modules introduce architectural extensions, their overall computational overhead remains reasonable and well-controlled, reflecting a deliberate balance between representational power and computational efficiency. Specifically, the FRBlock and DFCN modules exhibit substantially lower parameter counts (33.31 K and 26.9 K, respectively) and FLOPs (135.8 M and 110.1 M) compared to the original VGGBlock in U-Net++ (74.1 K parameters, 304.1 M FLOPs), with memory usage limited to 8.50 MB and 23.50 MB, respectively, thus demonstrating their lightweight nature. In contrast, the EAP module, which integrates ASPP and EMA mechanisms to enhance global context modeling, incurs a slightly higher computational cost (250 K parameters, 950 M FLOPs, and 31.0 MB memory usage). Nevertheless, it is worth noting that EAP is applied only once at the final stage of the encoder, and its resource consumption is localized and therefore manageable in the context of the entire network.

In summary, the design of each module in this study enhances structural awareness and improves fine-grained boundary modeling accuracy while maintaining low resource consumption, thereby achieving a favorable trade-off between efficiency and performance. These results demonstrate the lightweight and efficient nature of the proposed architecture, highlighting its potential for practical deployment in clinical settings.

To further assess the overall computational complexity and inference efficiency of the proposed model, Table 12 provides a comprehensive summary of resource consumption and performance metrics for each module integrated into the full U-Net++ architecture. The evaluation includes key indicators such as the number of parameters (Params), floating-point operations (FLOPs), average inference time per image, and peak VRAM usage. All experiments were conducted in a standardized environment using an NVIDIA RTX 3090 GPU to ensure fair performance comparisons and reproducibility.

Although the FRBlock module slightly increases inference time (8.82 ms), it substantially reduces the number of parameters (4.68 M) and FLOPs (19.51 G) compared to the original U-Net++ (9.16 M, 34.90 G), while maintaining stable segmentation performance (Dice: 82.74%). The EAP and DFCN modules further enhance feature representation capacity with inference times of 6.24 ms and 9.55 ms, respectively, and parameter counts of 18.12 M and 5.90 M. Additionally, both modules exhibit minimal memory overhead, with peak memory usage controlled at 192.02 MB and 168.10 MB.

Notably, even with the integration of all structural enhancement modules, the overall complexity of FED-UNet++ remains within acceptable limits, with a total parameter count of 18.82 M, FLOPs of 42.75 G, and an inference time of 11.91 ms. Under a peak VRAM usage of 198.37 MB, the model achieves optimal segmentation performance (IoU: 74.95%, Dice: 84.43%), demonstrating a well-balanced trade-off between computational efficiency and segmentation accuracy.

## 5. Conclusions

This paper proposes FED-UNet++, an improved network framework for hippocampus segmentation, designed to support the early diagnosis of Alzheimer’s disease (AD). Built upon the classical U-Net++ architecture, the model integrates three key innovations: the residual feature reconstruction block (FRBlock) for enhancing shallow feature representation, the efficient attention pyramid (EAP) for strengthening deep semantic modeling, and the dynamic frequency context network (DFCN) for integrating frequency-domain modeling with contextual awareness. These components jointly enhance the model’s ability to delineate complex hippocampal boundaries and significantly improve segmentation performance in medical imaging, particularly in neurodegenerative disease scenarios characterized by blurred and deformable lesion boundaries.

Extensive ablation studies and visual comparisons confirm the effectiveness of each component. FED-UNet++ demonstrates notable improvements in IoU, Dice, and other evaluation metrics on the publicly available Kaggle hippocampus dataset. Furthermore, it exhibits strong generalization capability on the 2D slice version of the 3D Task04_Hippocampus dataset, outperforming representative models such as DeepLabv3+, PSPNet, and SwinUNet, while maintaining low computational cost and high segmentation accuracy.

Although promising, several directions for future work remain:

1. Transitioning from 2D slices to 3D volumetric modeling could enable better representation of inter-slice continuity through 3D convolutions.
2. Clinical validation using real-world and multi-center MRI datasets is needed to evaluate the model’s robustness under diverse imaging conditions.
3. Diagnostic integration may allow for automated hippocampal volumetry and disease stage prediction, further extending the framework toward intelligent clinical decision support systems.
4. Deployment optimization through pruning, quantization, and lightweight architectural design can facilitate real-time inference on edge devices and within practical medical environments.

## Figures and Tables

**Table 1:** Sensitivity analysis of loss weight ratio (BCE:Dice) on segmentation performance.

BCE:Dice	IoU	Dice	Recall	Precision	HD95
0.7:0.3	74.83%	84.31% ± 0.23%	85.11%	85.67%	2.12
0.5:0.5	74.95%	84.43% ± 0.21%	85.31%	85.81%	2.06
0.3:0.7	74.74%	84.25% ± 0.24%	85.08%	85.59%	2.14

**Table 2:** Comparison experiments.

Datasets	Method	IoU	Dice	Recall	Precision	Accuracy	HD95
Kaggle dataset	U-Net	66.60%	79.20%	77.01%	82.33%	99.85%	6.1837
	U-Net++	70.51%	81.62% ± 0.38%	80.96%	83.71%	99.87%	4.7924
	SwinUNet	60.75%	73.56%	73.03%	75.78%	99.82%	5.8372
	PSPNet	51.70%	68.15%	72.40%	64.39%	99.75%	7.2048
	DeepLabv3+	69.89%	82.29%	89.70%	75.99%	99.86%	3.5913
	Ours	74.95%	84.43% ± 0.21%	85.31%	85.81%	99.89%	2.0611

**Table 3:** Ablation experiments.

Datasets	Method	IoU	Dice	Recall	Precision	Accuracy	HD95
Kaggle dataset	Baseline (U-Net++)	70.51%	81.62% ± 0.38%	80.80%	84.58%	99.87%	4.7924
	FR + U-Net++	72.10%	82.74% ± 0.36%	82.10%	85.21%	99.88%	3.4372
	EA + U-Net++	72.29%	82.91% ± 0.33%	84.37%	83.40%	99.88%	3.0167
	DF + U-Net++	72.45%	83.02% ± 0.31%	83.51%	84.47%	99.88%	2.4452
	FR + EA + U-Net++	73.18%	83.88% ± 0.27%	83.26%	85.37%	99.88%	2.5823
	FR + DF + U-Net++	73.08%	84.01% ± 0.25%	84.17%	84.57%	99.88%	2.3714
	EA + DF + U-Net++	73.37%	84.17% ± 0.23%	83.32%	85.79%	99.89%	2.2259
	FED-UNet++	74.95%	84.43% ± 0.21%	85.31%	85.81%	99.89%	2.0611

**Table 4:** Ablation experiment visualization. The red boxes highlight regions (hippocampal tail and connection areas) where FED-UNet++ achieves more accurate segmentation compared to the baseline.

Original	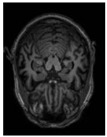	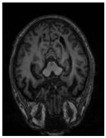	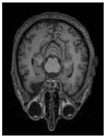	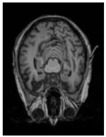	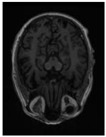
GT	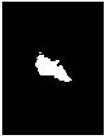	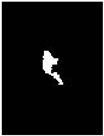	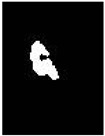	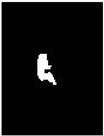	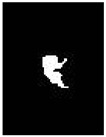
Baseline	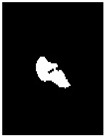	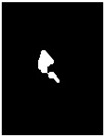	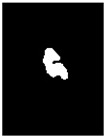	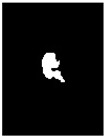	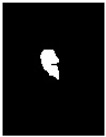
FR+	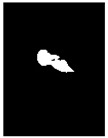	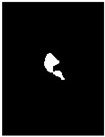	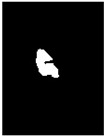	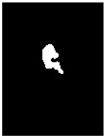	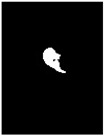
EA+	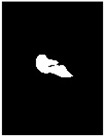	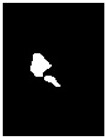	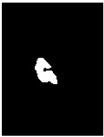	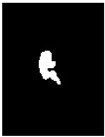	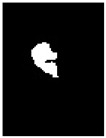
DF+	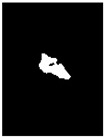	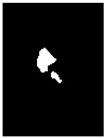	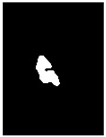	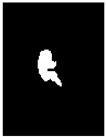	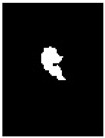
FR + EA+	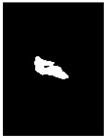	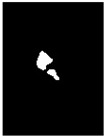	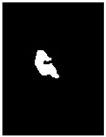	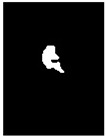	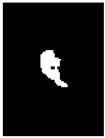
FR + DF+	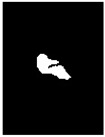	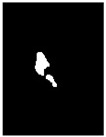	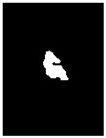	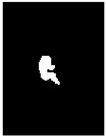	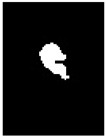
EA + DF+	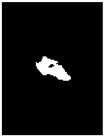	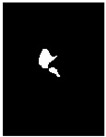	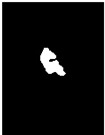	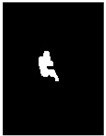	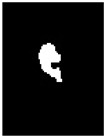
FED-UNet++ (FR + EA + DF+)	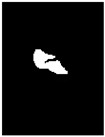	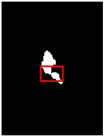	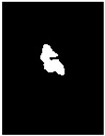	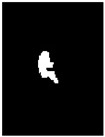	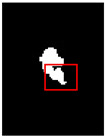

**Table 5:** Representative visualizations of attention and frequency responses from EAP and DFCN modules in the hippocampal region.

Original	GT	Representative Attention Activation Map from the EAP Module	Representative Frequency Response Visualization from the DFCN Module
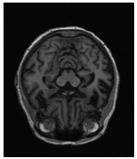	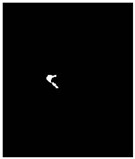	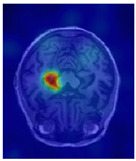	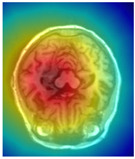

**Table 6:** Prediction failure cases of FED-UNet++ in hippocampus segmentation tasks.

Original	GT	Predict
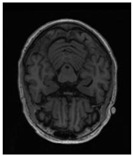	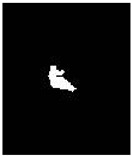	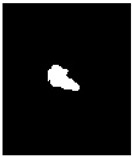
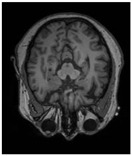	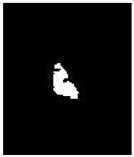	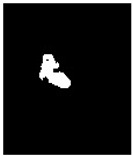

**Table 7:** Visual comparison of segmentation results before and after Gaussian noise interference.

Model: FED-UNet++	Original	GT	Segmentation Result
Before noise addition	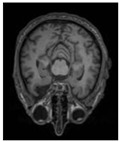	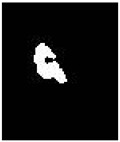	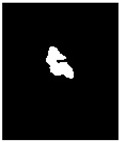
After adding noise	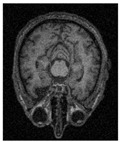	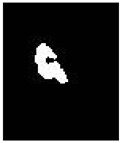	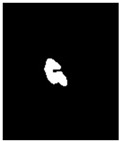

**Table 8:** Quantitative evaluation of segmentation performance before and after Gaussian noise addition.

Model: FED-UNet++	IoU	Dice	Recall	Precision	HD95
Before noise addition	74.95%	84.43% ± 0.21%	85.31%	85.81%	2.0611
After adding noise	72.63%	82.73 ± 0.33%	83.25%	82.57%	2.8088

**Table 9:** Generalization experiments.

Datasets	Method	IoU	Dice	Recall	Precision	Accuracy	HD95
Task004_ Hippocamps	Baseline	79.23%	88.40% ± 0.41%	87.81%	88.74%	98.40%	4.7285
	UNet	79.04%	88.02%	86.93%	89.42%	98.47%	4.8192
	PSPNet	76.98%	86.53%	87.04%	86.62%	98.27%	5.4796
	DeepLabv3+	78.44%	87.69%	87.80%	87.87%	98.33%	4.8951
	SwinUNet	77.65%	87.51%	86.02%	88.55%	98.26%	5.0143
	FR+	80.64%	88.89% ± 0.33%	88.67%	89.74%	98.51%	3.8629
	FR+EA+	81.78%	89.55% ± 0.30%	89.23%	90.67%	98.61%	3.2074
	Ours	82.51%	90.12% ± 0.27%	89.86%	90.94%	98.65%	2.8826

**Table 10:** Generalization experiment visualization.

Original	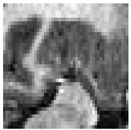	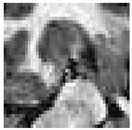	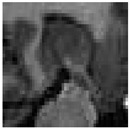	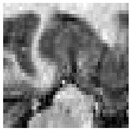
GT	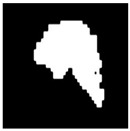	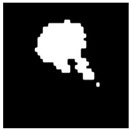	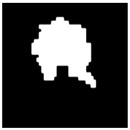	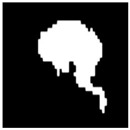
Baseline	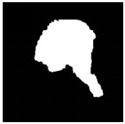	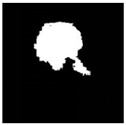	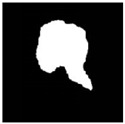	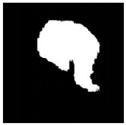
UNet	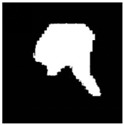	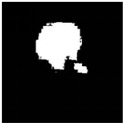	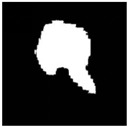	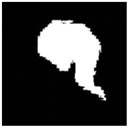
PSPNet	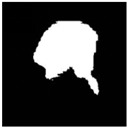	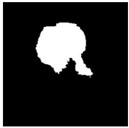	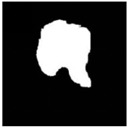	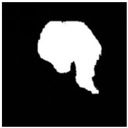
DeepLabv3+	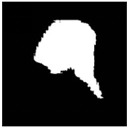	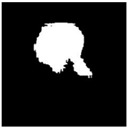	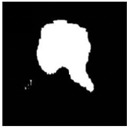	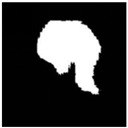
SwinUNet	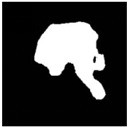	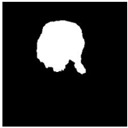	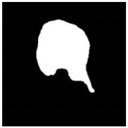	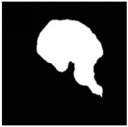
FR+	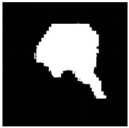	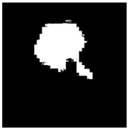	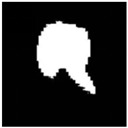	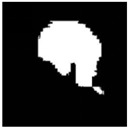
FR+EA+	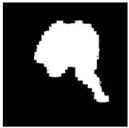	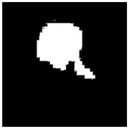	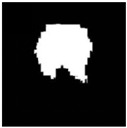	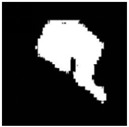
Ours	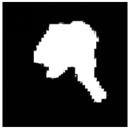	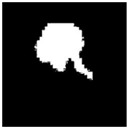	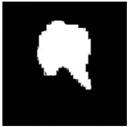	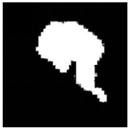

**Table 11:** Module-level resource overhead comparison (under the same channel configuration).

Block	Kernel Config	Channels (In/Out)	Params (M)	FLOPs (G)	Memory (MB)
VGGBlock (U-Net++)	3 × 3 + 3 × 3	64/64	0.0741	0.3041	6.08
FRBlock	3 × 3 + 1 × 1 + 3 × 3 + 1 × 1	64/64	0.0331	0.1358	8.50
EAP	ASPP + EMA	64/64	0.2500	0.9500	31.00
DFCN	1 × 1 + 3 × 3 + FFT + 1 × 1	64/64	0.0269	0.1101	23.50

**Table 12:** Overall model complexity and segmentation performance after integrating different modules into U-Net++.

Model	Params (M)	FLOPs (G)	Inference Time (ms)	Peak VRAM	IoU (%)	Dice (%)
U-Net++ (baseline)	9.16	34.90	5.33	157.38 MB	70.51	81.62 ± 0.38
FRBlock	4.68	19.51	8.82	155.47 MB	72.10	82.74 ± 0.36
EAP	18.12	37.21	6.24	192.02 MB	72.29	82.91 ± 0.33
DFCN	5.90	20.35	9.55	168.10 MB	72.45	83.02 ± 0.31
FED-UNet++	18.82	42.75	11.91	198.37 MB	74.95	84.43 ± 0.21

## Data Availability

The datasets used in this study are publicly available. The Kaggle hippocampus segmentation dataset can be accessed at https://www.kaggle.com/ (accessed on 26 June 2025), and the Task004_Hippocampus dataset from the Medical Segmentation Decathlon is available at http://medicaldecathlon.com/ (accessed on 30 June 2025).

## References

[1] Liu H., Gamboa H., Schultz T. (2023). Sensor-Based Human Activity and Behavior Research: Where Advanced Sensing and Recognition Technologies Meet. Sensors.

[2] Liu H., Gamboa H., Schultz T. (2024). Human Activity Recognition, Monitoring, and Analysis Facilitated by Novel and Widespread Applications of Sensors. Sensors.

[3] Alzheimer’s Association (2024). 2024 Alzheimer’s disease facts and figures. Alzheimers Dement..

[4] Frisoni G.B., Fox N.C., Jack C.R., Scheltens P., Thompson P.M. (2010). The clinical use of structural MRI in Alzheimer disease. Nat. Rev. Neurol..

[5] Mueller S.G., Weiner M.W., Thal L.J., Petersen R.C., Jack C.R., Jagust W., Trojanowski J.Q., Toga A.W., Beckett L. (2005). Ways toward an early diagnosis in Alzheimer’s disease: The Alzheimer’s Disease Neuroimaging Initiative (ADNI). Alzheimers Dement..

[6] Cuingnet R., Gerardin E., Tessieras J., Auzias G., Lehéricy S., Habert M.O., Chupin M., Benali H., Colliot O., Alzheimer’s Disease Neuroimaging Initiative (2011). Automatic classification of patients with Alzheimer’s disease from structural MRI: A comparison of ten methods using the ADNI database. Neuroimage.

[7] Litjens G., Kooi T., Bejnordi B.E., Setio A.A.A., Ciompi F., Ghafoorian M., van der Laak J.A.W.M., van Ginneken B., Sánchez C.I. (2017). A survey on deep learning in medical image analysis. Med. Image Anal..

[8] Ronneberger O., Fischer P., Brox T. (2015). U-Net: Convolutional Networks for Biomedical Image Segmentation. arXiv.

[9] Zhou Z., Siddiquee M.M.R., Tajbakhsh N., Liang J., Stoyanov D., Taylor Z., Carneiro G., Syeda-Mahmood T. (2018). UNet++: A Nested U-Net Architecture for Medical Image Segmentation. Deep Learning in Medical Image Analysis and Multimodal Learning for Clinical Decision Support, Proceedings of the 4th International Workshop, DLMIA 2018, and 8th International Workshop, ML-CDS 2018, Held in Conjunction with MICCAI 2018, Granada, Spain, 20 September 2018.

[10] Zhang Z., Liu Q., Wang Y. (2018). Road Extraction by Deep Residual U-Net. IEEE Geosci. Remote Sens. Lett..

[11] Oktay O., Schlemper J., Le Folgoc L., Lee M., Heinrich M., Misawa K., Mori K., McDonagh S., Hammerla N.Y., Kainz B. (2018). Attention U-Net: Learning Where to Look for the Pancreas. arXiv.

[12] Woo S., Park J., Lee J.Y., Kweon I.S., Ferrari V., Hebert M., Sminchisescu C., Weiss Y. (2018). CBAM: Convolutional Block Attention Module. Computer Vision—ECCV 2018, Proceedings of the 15th European Conference, Munich, Germany, 8–14 September 2018.

[13] Chen L.-C., Papandreou G., Kokkinos I., Murphy K., Yuille A.L. (2018). DeepLab: Semantic Image Segmentation with Deep Convolutional Nets, Atrous Convolution, and Fully Connected CRFs. IEEE Trans. Pattern Anal. Mach. Intell..

[14] Chen J., Lu Y., Yu Q., Luo X., Adeli E., Wang Y., Lu L., Yuille A.L., Zhou Y. (2021). TransUNet: Transformers Make Strong Encoders for Medical Image Segmentation. arXiv.

[15] He K., Zhang X., Ren S., Sun J. Deep Residual Learning for Image Recognition. Proceedings of the 2016 IEEE Conference on Computer Vision and Pattern Recognition (CVPR).

[16] Ouyang D., Deng Y., Wu Y., Li C., Jin L., Sun Y. Efficient Multi-Scale Attention Module with Cross-Spatial Learning. Proceedings of the 2023 IEEE International Conference on Acoustics, Speech and Signal Processing (ICASSP).

[17] Kong L., Dong J., Tang J., Yang M.-H., Pan J. (2025). Efficient Visual State Space Model for Image Deblurring. arXiv.

[18] Malekzadeh S. (2019). MRI Hippocampus Segmentation. Kaggle. https://www.kaggle.com.

[19] Simpson A.L., Antonelli M., Bakas S., Bilello M., Farahani K., van Ginneken B., Kopp-Schneider A., Landman B.A., Litjens G., Menze B. (2019). A Large Annotated Medical Image Dataset for the Development and Evaluation of Segmentation Algorithms. arXiv.

[20] Zhang D., Shen D., Alzheimer’s Disease Neuroimaging Initiative (2012). Multi-modal multi-task learning for joint prediction of multiple regression and classification variables in Alzheimer’s disease. Neuroimage.

[21] Sørensen L., Igel C., Pai A., Balas I., Anker C., Lillholm M., Nielsen M., Alzheimer’s Disease Neuroimaging Initiative, Australian Imaging Biomarkers and Lifestyle Flagship Study of Ageing (2016). Differential diagnosis of mild cognitive impairment and Alzheimer’s disease using structural MRI cortical thickness, hippocampal shape, hippocampal texture, and volumetry. Neuroimage Clin..

[22] Long J., Shelhamer E., Darrell T. Fully Convolutional Networks for Semantic Segmentation. Proceedings of the 2015 IEEE Conference on Computer Vision and Pattern Recognition (CVPR).

[23] Zhao H., Shi J., Qi X., Wang X., Jia J. Pyramid Scene Parsing Network. Proceedings of the IEEE Conference on Computer Vision and Pattern Recognition (CVPR), 2017.

[24] Chen L.C., Zhu Y., Papandreou G., Schroff F., Adam H. Encoder-Decoder with Atrous Separable Convolution for Semantic Image Segmentation. Proceedings of the European Conference on Computer Vision (ECCV), 2018.

[25] Hatamizadeh A., Nath V., Tang Y., Yang D., Myronenko A., Landman B., Roth H.R. UNETR: Transformers for 3D medical image segmentation. Proceedings of the IEEE/CVF Winter Conference on Applications of Computer Vision (WACV), 2022.

[26] Cao H., Wang Y., Chen J., Jiang D., Zhang X., Tian Q., Wang M. (2021). Swin-Unet: Unet-like pure transformer for medical image segmentation. arXiv.

[27] Wang J., Li X., Ma Z. (2025). Multi-Scale Three-Path Network (MSTP-Net): A New Architecture for Retinal Vessel Segmentation. Measurement.

[28] Zhao Y., Li X., Zhou C., Peng H., Zheng Z., Chen J., Ding W. (2024). A Review of Cancer Data Fusion Methods Based on Deep Learning. Inf. Fusion.

[29] Huang K.W., Yang Y.R., Huang Z.H., Liu Y.Y., Lee S.H. (2023). Retinal Vascular Image Segmentation Using Improved UNet Based on Residual Module. Bioengineering.

[30] Xing R. (2025). FreqU-FNet: Frequency-Aware U-Net for Imbalanced Medical Image Segmentation. arXiv.

[31] Hayat M., Aramvith S., Bhattacharjee S., Ahmad N. (2025). Attention GhostUNet++: Enhanced Segmentation of Adipose Tissue and Liver in CT Images. arXiv.

[32] Hayat M., Gupta M., Suanpang P., Nanthaamornphong A. Super-Resolution Methods for Endoscopic Imaging: A Review. Proceedings of the 2024 12th International Conference on Internet of Everything, Microwave, Embedded, Communication and Networks (IEMECON).

[33] Liang T.Y., Zhao H., Wang X., Chen Q., Lin Y., Zhang D. Integrating Vision Transformer with UNet++ for Hippocampus Segmentation in Alzheimer’s Disease. Proceedings of the 2024 46th Annual International Conference of the IEEE Engineering in Medicine and Biology Society (EMBC).

[34] Helaly H.A., Badawy M., Haikal A.Y. (2022). Toward Deep MRI Segmentation for Alzheimer’s Disease Detection. Neural Comput. Appl..

[35] Sanjay V., Swarnalatha P. (2024). Dominant Hippocampus Segmentation with Brain Atrophy Analysis-Based AD Subtype Classification Using KLW-RU-Net and T1FL. Alex. Eng. J..

[36] Alzheimer’s Disease Neuroimaging Initiative (2023). ADNI Data. https://adni.loni.usc.edu/.

